# Efficacy of Simple Short-Term *in Vitro* Assays for Predicting the Potential of Metal Oxide Nanoparticles to Cause Pulmonary Inflammation

**DOI:** 10.1289/ehp.11811

**Published:** 2008-09-17

**Authors:** Senlin Lu, Rodger Duffin, Craig Poland, Paul Daly, Fiona Murphy, Ellen Drost, William MacNee, Vicki Stone, Ken Donaldson

**Affiliations:** 1 University of Edinburgh, Edinburgh, UK; 2 School of Environmental and Chemical Engineering, Shanghai University, Shanghai, China; 3 Napier University, Edinburgh, UK

**Keywords:** electron paramagnetic resonance, EPR, inflammation, lungs, nanoparticles, oxidative stress, ROS

## Abstract

**Background:**

There has been concern regarding risks from inhalation exposure to nanoparticles (NPs). The large number of particles requiring testing means that alternative approaches to animal testing are needed.

**Objectives:**

We set out to determine whether short-term *in vitro* assays that assess intrinsic oxidative stress potential and membrane-damaging potency of a panel of metal oxide NPs can be used to predict their inflammogenic potency.

**Methods:**

For a panel of metal oxide NPs, we investigated intrinsic free radical generation, oxidative activity in an extracellular environment, cytotoxicity to lung epithelial cells, hemolysis, and inflammation potency in rat lungs. All exposures were carried out at equal surface area doses.

**Results:**

Only nickel oxide (NiO) and alumina 2 caused significant lung inflammation when instilled into rat lungs at equal surface area, suggesting that these two had extra surface reactivity. We observed significant free radical generation with 4 of 13 metal oxides, only one of which was inflammogenic. Only 3 of 13 were significantly hemolytic, two of which were inflammogenic.

**Conclusions:**

Potency in generating free radicals *in vitro* did not predict inflammation, whereas alumina 2 had no free radical activity but was inflammogenic. The hemolysis assay was correct in predicting the proinflammatory potential of 12 of 13 of the particles examined. Using a battery of simple *in vitro* tests, it is possible to predict the inflammogenicity of metal oxide NPs, although some false-positive results are likely. More research using a larger panel is needed to confirm the efficacy and generality of this approach for metal oxide NPs.

Nanotechnologies have the potential to improve many aspects of our life. Nanomaterials are either being used or have the potential to be widely incorporated in a range of applications, including textiles, finishes, and electronics, as well as having a spectrum of uses in medical imaging, disease diagnoses, and drug delivery. This potential is a result of unique physicochemical characteristics apparent at the nanoscale, such as large surface area, altered electronic properties, surface reactivity, and surface derivatization (Gwinn and Vallyathan 2006; Nel et al. 2006). However, concerns have been expressed regarding potential adverse effects of nanoparticles (NPs) on human and environmental health. In addition, NPs present in environmental air, derived from combustion sources, are believed to have adverse effects (Donaldson et al. 2005). Therefore, there have been calls to assess NPs for adverse human and ecologic toxicity before their widespread industrial application (Maynard et al. 2006; Royal Society and Royal Academy of Engineering 2004). However, many NP types are currently in use in industry and have been for decades, for example, titanium dioxide (TiO_2_) and carbon black (CB). Because of the large and growing numbers of untested NPs and their variants with actual or potential use in industry, there is a need for predictive approaches to assess their potential toxicity. In our previous studies, we demonstrated that ultrafine particles induce a greater amount of oxidative stress and inflammatory response than do fine particles of the same material (Li et al. 1999; Renwick et al. 2004). Reactive oxygen species have been hypothesized as key players of NPs’ mechanism of toxicity, and formation of free radicals by particles is considered to play a key role (Ayres et al. 2008).

We undertook the present study using a panel of metal oxide NPs, chosen because such particles are in high-volume industrial use and there is considerable potential for exposure. We hypothesized that simple toxicity assays and assays of free-radical–generating potential and oxidative stress might predict the inflammatory potential in rat lung caused by the same particles. Our results clearly reveal that a variety of short-term *in vitro* assays predict inflammogenicity of metal oxide NPs to varying extents but that hemolysis, a very simple assay, was the best predictor.

## Materials and Methods

### Particles

We obtained all chemical reagents from Sigma-Aldrich Company Ltd. (Gillingham, Dorset, UK) unless otherwise stated. Table 1 lists the size and surface area of NPs, and the commercial companies that supplied them.

Our panel comprised of 12 NPs plus alumina 3, which we included as a micro particle control (0.3 μm diameter); the panel also included a CB NP that was not a metal oxide. However, for convenience, we refer to the panel as the “metal oxide NP” panel and treat all the particles together in testing and analysis.

### Particle preparation

Because of their character, NPs tend to aggregate to some degree. We prepared NP stock solutions in deionized water (Milli-Q Academic, Millipore, MA, USA) (resistivity, 18.4 ΩM) or cell culture medium containing fetal calf serum, which acts as a dispersant, depending on assay. The solutions were sonicated for 5 min using a sonicator probe (220–240 V, 10.3 A Philip Harris Scientific, Lichfield, UK) to break up aggregation before dilution to different surface area doses, calculated from NP surface area.

### Electron paramagnetic resonance assay for free radical generation

We prepared NPs at a surface area dose of 3,000 cm^2^/mL in deionized water (Millipore; resistivity, 18.4 ΩM) as stock solution before dilution to 1,500 cm^2^/mL and 300 cm^2^/mL for electron paramagnetic resonance (EPR) investigation. Generation of free radicals from NP suspensions was studied in the presence of the spin trap Tempone-H (Alexis Co., Bingham, UK). We mixed 10 μL 100 mM Tempone-H in EDTA with 990 μL NP suspension in deionized water in an Eppendorf tube. We incubated the mixture at 37°C and transferred it immediately to a 50-μL glass capillary, which we then placed in a Miniscope MS200 EPR spectrometer (Magnettech, Berlin, Germany). Deionized water and H_2_O_2_ (100 μM) were selected as negative and positive controls, respectively. We recorded the EPR spectra at room temperature using the following instrumental conditions: microwave frequency, 9.39 GHz; magnetic field, 3,360 G; sweep width, 100G; scan time, 30 sec; number of scans, 3; modulation amplitude, 1.8G; receiver gain, 1,000.

### Cell-free dichlorofluorescein assay

To evaluate the oxidative potential of the NP fraction, we used the method described by Foucaud et al. (2007), modified for this study. Briefly, we chemically hydrolyzed 25 mL 1 mM 2’,7’-dichlorofluorescein diacetate (DCFH-DA) to 2’,7’-dichlorofluorescein (DCFH) at pH 7.0 with 100 μL 0.01 N NaOH in each well of a 48-well plate. The plate was put in the dark for 30 min at room temperature. We stopped the chemical reaction by adding 375 μL 0.1 M phosphate-buffered saline (PBS) and placing the plate on ice and then rapidly assayed it. Fifty μL of different NP solutions at surface area doses of 1,500 cm^2^/mL and 300 cm^2^/mL was added into the wells to obtain a final concentration of 10 μM DCFH. We added the NP solutions in triplicate and selected 4 U/mL horseradish peroxidase (HRP) and a blank solution (a mixture of 0.5 mL methanol, 2 mL 0.01 NaOH, and 7.5 mL 0.1 M PBS) as positive and negative controls, respectively. The fluorescence generated by the DCFH oxidation was measured immediately by use of a microplate reader at 485 nm excitation and 530 nm emission both with a slit width of 10 nm under constant agitation. Plates were read at different time points (1, 10, 20, 30, 60 min) and data were recorded as arbitrary fluorescence units.

### Cytotoxicity

We assessed cytotoxicity by lactate dehydrogenase (LDH) release. A549 lung epithelial cells were seeded in 24-well flat-bottomed culture plates with a density of 70,000 cells/well. When the cells grew to confluency in media containing 2% fetal bovine serum, they were exposed to a range of NP surface area doses (9.375–300 cm^2^/mL) for 24 hr. We measured LDH release according to the manufacturer’s instructions (Roche Diagnostics Ltd., West Sussex, UK) using pyruvic acid as a substrate. We determined LDH activity spectrophotometrically at 490 nm and expressed it as a percentage of total cellular LDH, which we measured in the cell lysates obtained by treatment with 0.1% Triton X-100. All experiments were carried out in triplicate and repeated three times.

### Hemolysis

We obtained erythrocytes from fresh human venous blood, drawn from healthy volunteer donors with informed oral consent, under the full institutional ethical approval of the University of Edinburgh. Ten milliliters of blood was added to 1mL 3.8% sodium citrate solution in a 15mL Falcon tube to prevent coagulation. We mixed the blood by gentle inversion of the tube and centrifuged it at 1,200 × *g* for 10 min. The plasma supernatant was discarded and the red blood cells (RBCs) were washed 4 times by suspending them in saline (0.9%) before centrifugation at 1,200 × *g* for 10 min. The final suspension consisted of 5% by volume RBC in saline. We prepared NP at a surface area dose of 300 cm^2^/mL in 0.9% saline and sonicated them for 10 min. NP stock solutions were diluted in 1:2 serial dilutions to 9.37 cm^2^/mL, and 150 μL NP suspensions were added, in duplicate, to a flat-bottomed 96-well plate. Negative control wells consisted of 150 μL saline, and positive control wells consisted of 150 μL 0.1% Triton-X 100. To each well, we added 75 μL RBC suspension and mixed gently by pipetting. The plates were incubated for 30 min at room temperature, shaking gently on an orbital plate shaker. After incubation, plates were centrifuged for 5 min and 75 μl carefully removed from each well and transferred to a clean plate. We determined the amount of hemoglobin released into the supernatant spectrophotometrically at a wavelength of 540 nm. The percent hemolysis was calculated using the equation of a straight line,


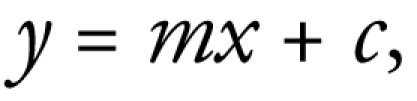


where % hemolysis (*x*) = [optical density (*y*) – negative control optical density (*c*)]/[(positive control optical density – negative control optical density)/100] (*m*).

### Instillation and bronchoalveolar lavage assessment

We used female Wistar rats, approximately 4 months old, throughout and carried out all handling, maintenance, and treatments under a specific license granted by the U.K. Home Office to one of the authors (KD) that ensures humane treatment and alleviation of suffering in all animal experiments. The animals were anesthetized with halothane, cannulated with a laryngoscope to expose the trachea, and 0.5 mL of different NP suspensions in saline were instilled into the lungs. All of the NPs were prepared at a surface area dose of 500 cm^2^/mL. We used animals instilled with 0.5 mL saline as controls. Animals were sacrificed 24 hr later with a single intraperitoneal injection of 2 mL sodium pentobarbitone (200 mg/mL). The trachea was cannulated with a luer port cannula (Portex, Smiths Medical International Ltd., Kent, UK), which was then tied in place. We removed the trachea and lungs by dissection and lavaged the lungs *in situ* with a single 8 mL volume of sterile saline. We retained this first bronchoalveolar lavage fluid (BALF) in a separate tube for analysis of biochemistry content (LDH and protein). Subsequently, we lavaged the lungs with a further three 8 mL volumes of sterile saline. All samples were centrifuged at 250 × *g* for 5 min at 4°C, the supernatant was removed, and the cell pellet from the first lavage was combined with the cells from the subsequent lavage before resuspension in 1 mL PBS. Cytospin preparations were made using 10,000 cells centrifuged at 15 × *g* for 3 min onto glass slides. The cells were fixed with methanol and stained using Diff Quick dyes (Raymond Lamb, Eastbourne, UK). We counted total cell numbers automatically with a nucleocounter (Chemomete, Allerød, Denmark). Three hundred cells per slide were counted and the results expressed as total number of polymorphonuclear neutrophils (PMNs) in the lung lavage.

### Statistical analysis

We conducted individual experiments in triplicate unless otherwise stated. Data are expressed as mean ± SE (*n* = 3) and were analyzed with GraphPad InStat software (Version 3, GraphPad Software, Inc., La Jolla, CA, USA). We determined statistical significance using one-way analysis of variance with post hoc Tukey's pairwise comparisons.

## Results

### EPR activity of the metal oxide NP panel

**Figure 1:**
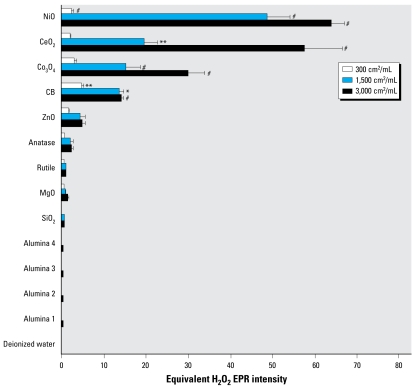
Intensity of free radicals formed by various surface area doses of a panel of NPs after incubation with the generic spin trap Tempone-H for 1 hr at 37°C. Values are means ± SD from three independent experiments. SiO_2_, silicon dioxide. Significance versus control: *p < 0.05, **p < 0.01, ^#^*p* < 0.001.

EPR results (Figure 1) showed responses dependent on surface area dose after NPs were incubated with the spin trap Tempone-H for 1 hr at 37°C. Where there was a detectable response, the free-radical intensity generated from all of the tested NP increased with surface area dose (correlation coefficient *R*^2^ = 0.994). Among the panel of NPs, free radical intensity was significantly increased only with nickel oxide (NiO), cerium dioxide (CeO_2_), cobalt(II,III) oxide (Co_3_O_4_), and CB.

### Fluorescence intensity of metal oxide NP panel by use of cell-free DCFH assay

**Figure 2:**
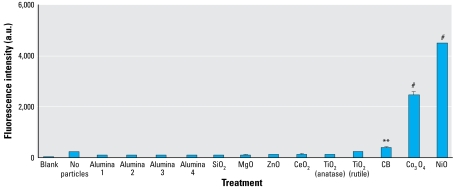
Fluorescence intensity [arbitrary units (a.u.)] of the panel of metal oxide NPs after incubation with DCFH for 1 hr at room temperature. The surface area dose of the NPs was 1,500 cm^2^/mL. Values are mean + SEM from three independent experiments. SiO_2_, silicon dioxide. Significance versus blank control: **p < 0.01. ^#^*p* < 0.001.

We investigated the oxidative ability of the metal oxide NP panel in a chemical assay of oxidative potential—the cell-free DCFH assay—at a surface area dose of 1,500 cm^2^/mL (Figure 2; all data shown are for 1 hr). The cell-free assay results showed time-dependent increases in fluorescence after NPs were incubated with DCFH (data not shown). The ranking of the oxidative potential effectively showed that only NiO, Co_3_O_4_, and CB had oxidizing activity against DCFH. In contrast, the remainder of the panel had very low oxidizing activity. It was especially notable that CeO_2_, which was a significant generator of free radicals in the EPR assay, showed no significant activity in the DCFH assay.

### Cytotoxicity of the metal oxide NP panel

To establish the cellular toxicity of the panel of NPs, we incubated alveolar epithelial A549 cells with NPs at different surface area doses (9.375–300 cm^2^/mL) for 24 hr and measured LDH release in cell lysates.

**Figure 3:**
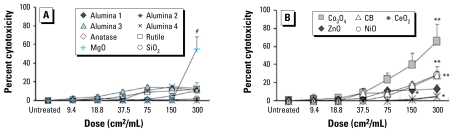
The LDH activity of the panel of NPs in alveolar epithelial A549 cell culture medium after 24-hr exposure at various surface area doses. (*A*) NPs that caused no significant cytotoxicity except for the anomalous high response seen with MgO at the highest dose. (*B*) NPs causing significant dose-related cytotoxicity. Values are mean ± SD from three independent experiments. Significance versus control: *p < 0.05, **p < 0.01, ^#^*p* < 0.001.

There was a clear surface area dose response with NiO, Co_3_O_4_, and CB (Figure 3A). Notably, the toxicity of CeO_2_ particles was insignificant. The cytotoxicities of the other metal oxide NPs, as expected from their low free radical and oxidizing activity against DCFH, were not significantly different from controls (Figure 3B). The response to magnesium oxide (MgO) was paradoxical in that cytotoxicity was negligible at lower surface area doses but was 55.3% at the highest dose, 300 cm^2^/mL.

### Hemolytic activity of the metal oxide NP panel

**Figure 4:**
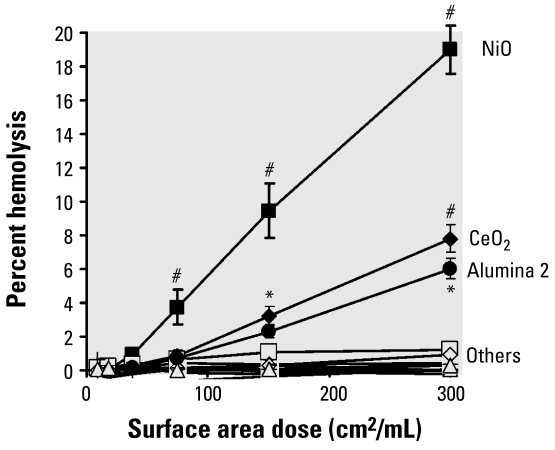
Hemolytic action of the members of the metal oxide NP panel. Values are mean ± SD from three independent experiments. Significance versus TiO_2_: *p < 0.05, ^#^*p* < 0.001.

Only three particles showed significant hemolytic activity, in the order of potency NiO > CeO_2_ = alumina 2 > > > all others (Figure 4).

### *Ability to cause inflammation* in vivo

**Figure 5:**
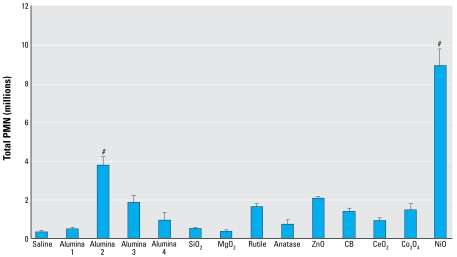
PMN numbers in lavage from groups of three rats instilled with metal oxide NPs at a surface area dose of 250 cm^2^/rat. Values are mean ± SD from three independent experiments. SiO_2_, silicon dioxide. Significance versus control: ^#^*p* < 0.001

In order to relate free radical activity of metal oxide NPs to their actual ability to cause lung inflammation, we instilled NPs into rats at a single surface area dose of 500 cm^2^/mL. We measured the inflammation potential by PMN numbers in BALF 24 hr after instillation. Only NiO_2_ and alumina 2 were significantly inflammogenic at the surface area dose used (Figure 5).

### Relationship between free radical activity and inflammation

**Figure 6:**
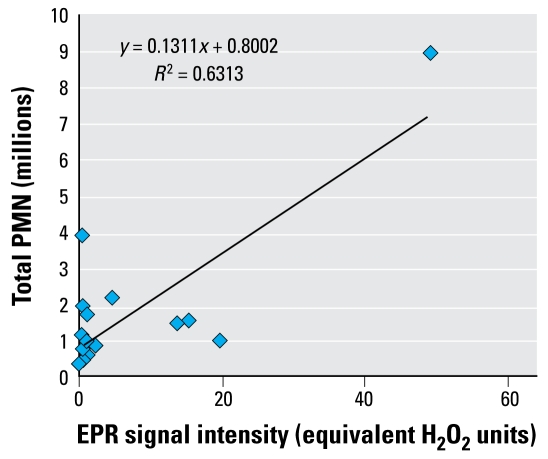
Relationship between free radical activity and ability to cause inflammation *in vivo*.

Figure 6 plots the free radical activity (EPR) (Figure 1) of the panel against the potential to cause inflammation (Figure 5), showing that the relationship is not good. In fact, most of the effect is from NiO, which is highly inflammogenic and generates large amounts of free radicals. Many particles have no free radical activity at all but generate moderate levels of inflammation (those against the vertical axis in Figure 6). Other particles show varying EPR signal intensities from 0 to 30 units but show consistently similar inflammogenicity in the range of 1–2 million PMNs.

### Relationship between oxidative activity in the DCFH assay and ability to cause inflammation

**Figure 7:**
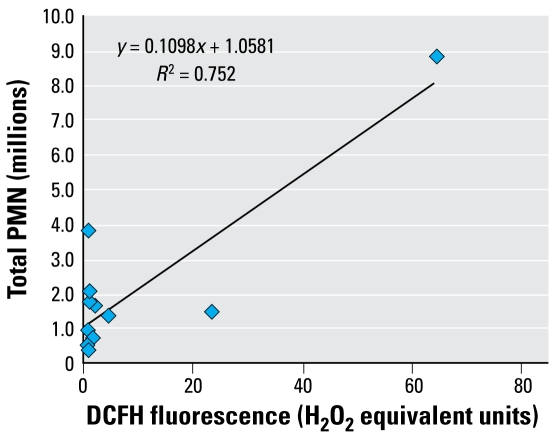
DCFH fluorescence plotted against inflammogenicity as measured by PMN number for the 1,500 cm^2^/mL surface area dose.

Figure 7 plots oxidative activity against DCFH (Figure 2) and ability to cause inflammation (Figure 5) for the 1,500 cm^2^/mL dose, showing that this relationship also is not good. Most of the particles had very little activity in the DCFH assay (those against the vertical axis in Figure 7) but have varying ability to cause inflammation, ranging from about 0.5 million to almost 4 million PMNs in the lavage. However, the NiO NP did show high oxidative potential and high inflammogenicity.

### Metal composition

Table 2 shows the water-soluble metal content of the NP panel. Most NPs had a low level of metal contamination, although the soluble forms of the metals from which the NPs were made [e.g., zinc (Zn) from zinc oxide (ZnO)] were present in larger quantities.

## Discussion

Concern regarding the potential toxicologic effects of NPs has been voiced in a number of publications (e.g., Borm et al. 2006; Donaldson and Stone 2007; Maynard et al. 2006). The large number of NP types and their variants, as a result of size, coatings, and other factors, poses a real problem for toxicologic testing, and it has been suggested that a structure–activity relationship (SAR) might be derived for NP (Oberdorster et al. 2005). Such a model, based on the type of SAR models used in chemical toxicology (Cronin et al. 2003), would allow the toxicity of any untested NP to be predicted from its structural and chemical characteristics. Such a SAR already exists in particle toxicology for one class of particles—high-aspect-ratio or fibrous particles, including asbestos. This SAR is based on length and ability to persist in the lungs (Donaldson and Tran 2004) and has proved to be robust across a number of fiber types.

Short-term testing *in vitro* provides another approach that might allow prediction of pathogenic potential and that may also contribute to developing a SAR. No single structural correlate is likely to control the various toxicities displayed by the large number of NP types that exist and need to be tested, but in restricting this study to metal oxides, we may have limited the different parameters that could dictate toxicity.

One structural property of NP that has been advanced to explain effects of NP at the cellular level is the ability of NPs to cause oxidative stress. A number of groups, including ours, have identified oxidative stress as a likely property that might cause adverse effects at the cellular level in cells exposed to NPs of various types (Ayres et al. 2008). Oxidative stress can lead to membrane damage, death, inflammation, or selective gene expression, depending on the dose (Nel et al. 2006). Donaldson and coworkers have reviewed the role of oxidative stress in the action of combustion-derived NPs (Donaldson et al. 2005) and manufactured NPs (Donaldson and Stone 2007). Nel et al. (2006) have advanced a model involving incremental severity of oxidative stress in triggering a cascade of responses. The potential to cause oxidative stress is a feature of many pathogenic particles (Donaldson et al. 1996), and oxidative stress in cells exposed to particles is recognized as a key pathogenic event that can lead to cell death, inflammation (Donaldson and Tran 2002), and genotoxicity (Schins 2002). Cellular oxidative stress could occur as a result of free radicals produced at the surface of the particles or by soluble components such as metals or organics (Donaldson et al. 2004) associated with NPs. This type of free radical activity, the type under study here, can be referred to as intrinsic free radical activity. Additionally, once inside cells, particles could cause oxidative stress by effects on cells that are not driven by intrinsic free radicals, such as effects that deplete antioxidants or stimulate mitochondrial respiration and indirectly cause oxidative stress (Ayres et al. 2008). In addition to testing for a structural correlate such as free radical activity, we chose two simple assays of particle toxicity: cytotoxicity measured by LDH release, and hemolysis. These are not factors that would directly contribute to a SAR but are simple assays that act as surrogates for structure and that would allow full elucidation of the role of structure in a SAR at a later date. For example, studying the structural correlate of hemolysis, if it was a good predictor of inflammation, would be much easier than studying the structural correlates for inflammation, which requires animals and considerable investment of time and money.

We therefore tested the hypothesis that the intrinsic free radical activity and membrane-damaging potential of a panel of metal oxide NPs, as measured by a battery of *in vitro* tests, was related to the ability to cause inflammation. We used four short-term assays *in vitro*, two of which assessed one structural correlate—the ability to generate free radical and oxidative stress—and two cell-based assays where toxicity was the sum of structural correlates that culminated in membrane damage/cell death.

Our previous research on insoluble particles and NP, which included the metal oxide titanium dioxide as well as CB, has demonstrated the importance of surface area as the dose metric. In inhalation studies, the onset of inflammation at high lung burden for two very different low-toxicity particles was at the same surface area dose and not the same mass dose (Tran et al. 2000). Instillation studies also highlighted the importance of surface area alone as the biologically effective dose for low-toxicity dusts and surface area times surface reactivity for insoluble particles with a reactive surface (Duffin et al. 2007). Donaldson et al. (2008) further demonstrated the key role of surface area by establishing concordance between *in vivo* inflammogenicity and proinflammatory gene expression in epithelial cells *in vitro*, when they used surface area doses of particles.

In this study we tested 11 metal oxide NPs because these represent a category of NPs that are in bulk use and to which a large number of people are likely to be exposed. We also included in our panel one of the metal oxides, alumina, as a larger microparticle (alumina 2) and NP CB that we have used extensively in the past. Thus, the final panel had 13 members. We purposely chose three different nanoaluminas to determine the possible variation in toxicity between the same nominal NP obtained from different sources and of different sizes.

The ability of NPs to aggregate is one of their best-documented properties, and human exposures will comprise exposures to NPs aggregated to some degree, so the use of aggregated NPs in toxicology studies is most relevant. We did not systematically control aggregation in this study, although we subjected all NPs to the same dispersion protocol. It is important to note that the increased inflammogenicity associated with increased NP surface area still occurs in aggregated NPs. For example, in the original inhalation study of Ferin et al. (1992), the exposures were to fine and ultrafine (NP) TiO_2_, but the airborne particle size was roughly the same for both (around 1 μm); therefore, the NP TiO_2_ was aggregated, because their primary particle size was about 20 nm. Nevertheless, the greater inflammogenicity of the NP TiO_2_ was evident and was consistent with its higher surface area, despite aggregation. In our own detailed analysis of the role of surface area in NP inflammogenicity (Duffin et al. 2007) using instillation of NPs, we injected a range of different low-toxicity, low-solubility NPs into rat lungs. Inevitably, the NPs were aggregated, but their inflammogenicity was exactly consistent with surface area, indicating that aggregation does not affect surface area dose (Duffin et al. 2007).

In the EPR assay for free radical generation, we used Tempone-H, a highly sensitive but nonspecific spin trap known to detect superoxide anion and peroxynitrite. The metal oxide panel we used here showed a range of intrinsic free radical activities, but only Co_3_O_4_, CeO_2_, NiO, and CB produced significant free radicals. Free radical generation has been described in other studies of NPs (Carrasco-Flores and Laverne 2007; Ionita et al. 2007; Kumar et al. 2006), but different protocols and spin traps have been used. The present study is the only one known to us that has used the exact same protocol and spin trap to test a range of NPs at equal surface area doses, allowing direct comparison among NPs regarding their intrinsic ability to generate free radicals.

We also measured the NPs’ ability to cause chemical oxidation of a target molecule, DCFH, as an alternative to EPR. The DCFH assay is easier to carry out and does not require an expensive EPR instrument; it needs only a fluorimeter, common in most laboratories. DCFH been used extensively to analyze intra-cellular oxidative stress caused by particles (Imrich and Kobzik 1998). DCFH is rapidly oxidized to fluorescent DCFH by hydroxyl radicals, peroxynitrite, and H_2_O_2_, the latter requiring the presence of a peroxidase such as HRP, as used here (Myhre et al. 2003). We saw significantly increased DCFH oxidation only with NiO, Co_3_O_4_, and CB. The ability of CB to cause DCFH oxidation has been reported previously (Foucaud et al. 2007).

There are a number of ways to assess oxidative potential, and each has advantages and disadvantages. We believe that a simple assay of oxidative potential is desirable because the gold standard of EPR is beyond the means of many labs. Fluorescent probes, including DCFH, have been used extensively for detecting particle-derived oxidative stress. This has been reviewed recently, and DCFH was found to efficiently detect oxidative stress from particles caused by a number of reactive species (Cohn et al. 2008). Because in this study we had no foregone conclusions regarding which species might be driving oxidative stress (e.g., superoxide anion, hydroxyl radical), we required a broad-spectrum sensor that detected different species.

We then compared the abilities of the various NPs to cause cytotoxicity using the LDH release assay. Adsorption of protein such as LDH onto the high surface area of NPs may create misleading results in this assay, but our results showed that NiO, CeO_2_, Co_3_O_4_, and CB, the same four NPs that generated significant intrinsic free radical activity, also caused significant cytotoxicity. Three out of four of the NPs that caused significant oxidation of DCFH were also significantly cytotoxic. We found an anomalous result with MgO, which had zero cytotoxicity at the first four doses but caused almost 60% cytotoxicity at the highest dose. We conclude that this is a nonphysiologic artifact possibly due to electrochemical imbalance affecting cell membrane integrity; we therefore exclude the high-dose result as irrelevant and do not discuss it further. Therefore, potential to generate free radical activity *in vitro* is relatively effective in predicting cytotoxicity *in vitro*, but not effective in predicting inflammogenicity *in vivo* (see below).

We further investigated the nature of particle/membrane interactions with a hemolysis assay, which detects direct damaging interactions between particle surfaces and membranes, which can be driven by oxidative stress for some particles (Razzaboni and Bolsaitis 1990) but can also occur when there is no direct evidence of a mechanism for free radical activity (Clouter et al. 2001). The hemolysis assay was positive for NiO, as expected, probably by an oxidative action. However, alumina 2 was also hemolytic, suggesting that it acted by a direct membranolytic or membrane-perturbing action to cause inflammation; based on results from our other assays, however, it is unlikely that the effect is caused by oxidative stress. CeO_2_ was also hemolytic, but did not cause inflammation.

We wished to relate all of these *in vitro* assay correlates of structure to the activity of lung inflammation. Only two NPs caused significant inflammation after instillation into rat lung—NiO_2_ and alumina 2. The demonstration that NiO_2_ was inflammogenic was consistent with the other data, showing it to be the most consistent and most potent generator of oxidative stress and intrinsic free radicals. The finding that alumina 2 was inflammogenic was unexpected, given the uniform negativity of all of the aluminas in all of the other assays. The similarity in levels of inflammation (between ~0.25 million and ~2.0 million PMNs) produced by most of the particles was consistent with the fact that these were low-toxicity particles (e.g., aluminas, TiO_2_, CB, MgO). We have previously found that surface area alone is the biologically effective dose controlling the extent of inflammation caused by low-toxicity, low-solubility particles after instillation into the lungs (Duffin et al. 2007). All these NPs were delivered at the same surface area dose.

It is notable that all of the particle treatments caused a transient low-level inflammation because of the high dose rate, but only two of these reached statistical significance. Although we demonstrated acute inflammation, the literature on particulate matter with aerodynamic diameters ≤ 2.5 μm suggests that the effects of combustion-derived NPs on the respiratory and cardiovascular systems can be acute—within a few hours or a day for cardiovascular effects (Peters et al. 2001), so transient inflammation, as studied, may have relevance.

We anticipated, from the model described by Duffin et al. (2007), that those NPs showing enhanced ability to generate free radicals (NiO, CeO_2_, Co_3_O_4_, CB) would all have extra reactivity and so would present a larger dose based on the hypothesis that the total dose equates to surface area times surface reactivity. However, this was not the case: only NiO fit this hypothesis; in addition, alumina 2 caused significantly more inflammation than other alumina NPs and more than would be predicted from its presumed status as a low-toxicity material and its low intrinsic free radical activity. Certainly the other two nanoalumina samples and the micrometer-sized alumina had no activity in any assay, and alumina 2 was distinctive for being the only alumina to cause lung inflammation.

When we measured the soluble (water-desorbable) metal released by each NP type, we found a relationship between the ranking for the amount of soluble metal released by any NP type and the activity in the assays, with the key exception of alumina 2. Transition metals were most common in the NPs generating large amounts of free radicals, but the overall levels were low.

Table 3 sums all of the data from the assays used in this study and shows that no single assay was 100% predictive of potential to cause inflammation *in vivo*, although the highest free radical generator also caused significant inflammation. However, one of the NPs that did not generate free radicals, alumina 2, also caused inflammation. NPs that caused free radical generation and oxidative stress were in general cytotoxic, but cytotoxicity was not well related to inflammation. This suggests that antioxidants and free radical scavengers are generally effective *in vivo* in the lungs.

Results for CeO_2_ and alumina 2 were paradoxical in that CeO_2_ was high in intrinsic free radical activity and was hemolytic but had no other activity, including no inflammation, whereas alumina 2 was inflammatory but had no free radical or other activity except for being positively hemolytic. CeO_2_ may have reacted with the spin trap to form an adduct that was not free radical mediated, which requires further study. We have extensively described elsewhere that CeO_2_ NPs have no ability to generate oxidative stress in other cell-free and cellular systems (Park et al. 2008).

Although oxidative stress was the major particle structure we considered here, we conclude that for this panel of metal oxide NPs, neither intrinsic free radical generation nor oxidative stress, assessed with DCFH, predicts ability to cause inflammation. NiO, the particle that was most oxidatively active as assessed by EPR, was inflammogenic, but three NPs (CB, Co_3_O_4_, and CeO_2_), all significantly free radical generating, were not significantly inflammogenic. One particle with no free radical, oxidative stress, or cytotoxic activity, alumina 2, was significantly inflammogenic. Using hemolysis assay of direct membrane-damaging potential, alumina 2 was positive, along with NiO; however, CeO_2_ was also positive in this assay but was not inflammogenic. Thus, the hemolysis assay indicated positive for three particles, two of which were inflammogenic, and was more effectively predictive than any other assay.

Table 4 sums the utility of the various assays for detecting inflammogenicity of the metal oxide NP panel. It shows that no single assay was completely predictive of inflammogenicity. It does show, however, that the hemolysis assay was a better predictor of potential to cause inflammation than any of the other assays: it was predictive for 12 of 13 of particles but gave a false positive for CeO_2_. It is important that this type of screening not produce false negatives, whereas later screening stages can deal with false positives. Hemolysis, which showed only a single false positive and no false negatives, rates well for this reason.

It is notable that most particles in the panel were of low toxicity, reflecting the general absence of large-scale occupational lung disease where such materials are used in bulk. However, it would be instructive to screen more high-toxicity particles before we can accept this model for predictive testing of metal oxide NPs. We do not know whether this model can be generalized to NPs other than metal oxides, and this should be tested. It is unlikely that fiber-type toxicity of high-aspect-ratio NPs would be testable in this paradigm, because this type of particle requires highly specific assays (Poland et al. 2008). The fact that one out of three nanoaluminas was inflammogenic points out the dangers of assuming that testing a representative NP from a class (e.g., aluminas) gives a representative result. More research is needed on the nature of the surface of alumina 2 in relation to the others, which should be illuminating regarding the mechanism of alumina 2 inflammogenicity.

There is a certain irony that in the era of “omics” one of the first assays to be used to detect particle toxicity as far back as 35 years ago (Harington 1972), hemolysis, emerges as a good predictor of inflammogenic potential of metal oxide NPs. Perhaps we should not be surprised that an assay that measures the interaction between the NPs and the membrane detects NP inflammogenic effects because these may be central to the inflammogenic action of these materials.

## Figures and Tables

**Table 1:** Size and surface area of the panel of metal oxide particles.

Nominal chemistry	CeO_2_	NiO	TiO_2_ rutile	TiO_2_ anatase	MgO	Co_3_O_4_	ZnO	Al_2_O_3_	Al_2_O_3_^a^	Al_2_O_3_^b^	Al_2_O_3_	SiO_2_	Carbon black^c^
Designated name	CeO_2_	NiO	Rutile	Anatase	MgO	Co_3_O_4_	ZnO	Alumina 1	Alumina 2	Alumina 3	Alumina 4	SiO_2_	CB
Size (nm)	20–30	10–20	30–40	5	20	20–30	90–210	2–4	7	300	20	10	14
Surface area (m^2^/g)	24	92	28	259	42	35	50	250	221	25	103	523	254
Mass (mg) per 500 cm^2^	20.8	5.4	17.9	1.9	11.9	14.3	10.0	2.0	2.3	20.0	4.9	1.0	2.0

SiO_2_, silicon dioxide. Alumina 3 is not an NP and that CB is not a metal oxide. All chemicals provided by Nanostructural and Amorphous Materials, Inc. (Houston, TX, USA) except as noted.

a KRAHN Chemie GmbH (Hamburg, Germany),

b BAIKOWSKI Chimie (Annecy, France),

c Evonik Degussa GmbH (Frankfurt, Germany).

**Table 2:** Soluble metal content of the panel of metal oxide NPs after desorption in water.

	Metal concentration (μg/g)										
NP	Cd	Co	Cr	Cu	Fe	Mn	Ni	Ti	V	Zn	Total
Alumina 1	< 0.1	0.4	< 0.1	0.4	0.7	0.1	0.2	< 0.1	0	0.3	2.1
Alumina 2	< 0.1	7	< 0.1	0.2	0.6	< 0.1	1.2	0.2	0	0	9.2
Alumina 3	< 0.1	0.2	< 0.1	0.5	0	< 0.1	< 0.1	< 0.1	0.1	1.4	2.2
Alumina 4	< 0.1	8.8	< 0.1	0.3	0	< 0.1	1.7	0.1	0	0.1	11
SiO_2_	< 0.1	0.5	< 0.1	0.1	0.8	< 0.1	17.5	1	0	0	19.9
MgO	< 0.1	0.1	0.1	0.4	0.4	0.1	< 0.1	< 0.1	0	0	1.1
Rutile	< 0.1	0.6	0.1	1.8	0.9	< 0.1	< 0.1	2943.2	3.8	23.8	2974.2
Anatase	< 0.1	54.2	< 0.1	1.6	0.6	< 0.1	2.2	0.0	0.0	0.0	58.6
ZnO	1.1	0.3	0.2	0.7	0	0.2	12.6	< 0.1	0	732.2	746.2
CB	< 0.1	0.3	< 0.1	0.2	0.1	< 0.1	2.6	0.2	0.1	0.1	3.6
Co_3_O_4_	< 0.1	1483.9	< 0.1	1.6	2.3	0.1	66.8	< 0.1	0	0.9	1555.6
CeO_2_	2.5	17.9	< 0.1	6.4	0	< 0.1	< 0.1	< 0.1	1.4	91.1	116.8
NiO	< 0.1	2.7	0.8	0.7	1.9	0.2	41353.4	4	0	175.7	41539.4

Abbreviations: Cd, cadmium; Co, cobalt; Cr, chromium; Cu, copper; Fe, iron; Mn, manganese; Ni, nickel; SiO_2_, silicon dioxide; Ti, titanium; V, vanadium.

**Table 3:** Summarized output of the assays for the panel of metal oxide particles.

	Assay					Rank for soluble metal content				
NP	EPR	Hemolysis	DCFH	Cytotoxicity	Inflammation	Fe	Cu	V	Zn	Total
Alumina 1	—	—	—	—	—	5	7	ND	8	12
Alumina 2	—	*	—	—	#	6	10	ND	ND	9
Alumina 3	—	—	—	—	—	ND	6	3 =	6	11
Alumina 4	—	—	—	—	—	ND	9	ND	9	8
SiO_2_	—	—	—	—	—	4	11	ND	ND	6
MgO	—	—	—	(*)	—	7	7	ND	ND	13
Rutile	—	—	—	—	—	3	2	1	4	2
Anatase	—	—	—	—	—	ND	ND	ND	5	7
ZnO	—	—	—	(—)	—	ND	4	ND	1	4
CB	#	—	**	**	—	8	10	3 =	10	10
Co_3_O_4_	#	—	#	**	—	1	3	ND	7	3
CeO_2_	#	#	—	*	—	ND	1	2	3	5
NiO	#	#	#	**	#	2	4	ND	2	1

Abbreviations: Cu, copper; Fe, iron; ND, not detectable; SiO_2_, silicon dioxide; V, vanadium. The numbers in the metal columns relate to ranking for soluble metals, with 1 the highest level. Asterisks denote the significance of the increase compared with controls for the highest doses used (EPR, hemolysis, cytotoxicity) or the single dose used (DCFH, inflammation) (see Figures 1–7). Parentheses for MgO in the cytotoxicity assay indicate marked cytotoxicity at the highest dose and no effect in any of the lower doses, which we conclude to be artifactual; parentheses for ZnO in the cytotoxicity assay indicate that the only significant effect was at the dose one step lower than the highest dose and that the highest dose was in fact not significantly increased compared with the control.

* *p* < 0.05,

** *p* < 0.01,

# *p* < 0.001.

**Table 4:** Efficacy of the various *in vitro* assays for predicting the inflammogenicity of metal oxide NPs.

*In vitro* assay	Total no. of NPs used in the assay	No. (%) NPs that were correctly predicted as inflammogenic/Non-inflammogenic	No. of NPs that were significantly positive in this assay	No.false positive (%)	No. false negative (%)
Hemolysis	13	12 (92)	3	1 (8)	0 (0)
EPR	13	9 (69)	4	3 (23)	1 (8)
DCFH	13	10 (77)	3	2 (15)	1 (8)
Cytotoxicity	13	9 (69)	4	3 (23)	1 (8)
